# CEACAM1 controls the EMT switch in murine mammary carcinoma *in vitro* and *in vivo*

**DOI:** 10.18632/oncotarget.11650

**Published:** 2016-08-27

**Authors:** Wegwitz Florian, Eva Lenfert, Daniela Gerstel, Lena von Ehrenstein, Julia Einhoff, Geske Schmidt, Matthew Logsdon, Johanna Brandner, Gisa Tiegs, Nicole Beauchemin, Christoph Wagener, Wolfgang Deppert, Andrea Kristina Horst

**Affiliations:** ^1^ Clinic for General, Visceral and Pediatric Surgery, University Medical Center Göttingen, Georg-August-University of Göttingen, D-37077 Göttingen, Germany; ^2^ Institute for Tumor Biology, University Medical Center-Hamburg-Eppendorf, D-20251 Hamburg, Germany; ^3^ Center for Diagnostics, University Medical Center Hamburg-Eppendorf, D-20251 Hamburg, Germany; ^4^ Pharmaceutical Institute, Christian-Albrechts-University Kiel, D-24118 Kiel, Germany; ^5^ Dermatology and Venerology Department and Clinic, University Medical Center Hamburg-Eppendorf, D-20251 Hamburg, Germany; ^6^ Institute for Experimental Immunology and Hepatology, University Medical Center Hamburg-Eppendorf, D-20251 Hamburg, Germany; ^7^ Goodman Cancer Research Centre and Departments of Biochemistry, Medicine and Oncology, McGill University, Montreal, H3G1Y6, Canada

**Keywords:** CEACAM1, Wnt-pathway, EMT, WAP-T, breast cancer

## Abstract

We analyzed the molecular basis for carcinoembryonic antigen-related cell adhesion molecule 1 (CEACAM1)-controlled inhibition of epithelial-mesenchymal transition (EMT) in a mouse model for mammary adenocarcinoma (WAP-T mice). We demonstrate that silencing of CEACAM1 in WAP-T tumor-derived G-2 cells induces epithelial-mesenchymal plasticity (EMP), as evidenced by typical changes of gene expression, morphology and increased invasion. In contrast, reintroduction of CEACAM1 into G-2 cells reversed up-regulation of genes imposing mesenchymal transition, as well as cellular invasion. We identified the Wnt-pathway as target for CEACAM1-mediated repression of EMT. Importantly, β-catenin phosphorylation status and transcriptional activity strongly depend on CEACAM1 expression: CEACAM1^high^ G-2 cells displayed enhanced phosphorylation of β-catenin at S33/S37/T41 and decreased phosphorylation at Y86, thereby inhibiting canonical Wnt/β-catenin signaling. We identified Src-homology 2 domain-containing phosphatase 2 (SHP-2) as a critical binding partner of CEACAM1 that could modulate β-catenin Y86 phosphorylation. Hence, CEACAM1 serves as a scaffold that controls membrane proximal β-catenin signaling. *In vivo*, mammary tumors of WAP-T/CEACAM1^null^ mice displayed increased nuclear translocation of β-catenin and a dramatically enhanced metastasis rate compared to WAP-T mice. Hence, CEACAM1 controls EMT *in vitro* and *in vivo* by site-specific regulation of β-catenin phosphorylation. Survival analyses of human mammary carcinoma patients corroborated these data, indicating that CEACAM1 is a prognostic marker for breast cancer survival.

## INTRODUCTION

Malignancies of the breast, along with colon cancer, cause the most frequent cancer-associated deaths in women. Although long-term survival has improved, 16.7% of breast cancer patients develop deadly metastatic disease [1]. Epithelial-to-mesenchymal transition (EMT) and mesenchymal-to-epithelial transition (MET) are principal tissue plasticity programs in embryogenesis, wound healing, fibrosis, cancer progression and metastasis [2]. This epithelial-mesenchymal plasticity (EMP) is a critical determinant of tumor invasion, stemness and chemoresistance of breast cancer cells, and its pivotal importance for disease outcome has been highlighted by numerous reports [3–9]. To further elucidate the molecular mechanisms that contribute to EMP and EMT, we used the well-characterized WAP-T mouse model that mimics the spontaneous development of mammary adenocarcinoma in humans [10–15]. In WAP-T mice, mammary glands develop normally. However, with the onset of lactation, the WAP promoter is specifically activated in epithelial cells of the mammary gland and drives expression of the simian virus 40 (SV40) large tumor antigen (LT), which compromises the tumor-suppressive activity of pRB and p53 [10, 16]. Invasive mammary carcinomas in WAP-T mice share features with human basal-like/triple-negative cancer [10, 13–15, 17]. Recently, we demonstrated that expression of mutant p53 in WAP-T tumors is mechanistically linked with EMP and enhanced metastasis [18, 19]. Interestingly, this aggressive tumor phenotype was inversely correlated with the expression of carcinoembryonic-related antigen cell adhesion molecule 1 (CEACAM1).

CEACAM1 is a member of the carcinoembryonic antigen (CEA) family in the superfamily of immunoglobulins. It is expressed on epithelial, endothelial or hematopoietic cells and its transcript undergoes extensive splicing, producing variants with or without a transmembrane domain, and long (CEACAM1-L) or short cytoplasmic tails (CEACAM1-S) [20]. CEACAM1 is involved in the regulation of metabolism, maintenance of tissue architecture and differentiation, tumor growth, innate immunity, and blood and lymphatic vessel growth and remodeling [21–25]. Recently, CEACAM1 has been identified as a crucial factor mediating the tolerance and exhaustion of immune cells through interaction of its variable (V)-like domain with that of T-cell immunoglobulin domain and mucin domain-3 (TIM-3) [20, 26–29]. Its role as a negative regulatory co-receptor also depends on an intact ITIM domain, which serves as a binding partner for Src family kinases and the Src-homology 2 domain-containing phosphatases (SHP) SHP-1 and SHP-2 ([20], and references therein). By means of CEACAM1-dependent scaffolding of signaling complexes, CEACAM1-L down-modulates the activity of receptor tyrosine kinases.

CEACAM1 expression levels are usually reciprocally connected to poor prognosis in many cancers including mammary carcinomas [30–36]. More specifically, CEACAM1 expression correlates with good prognosis in mammary carcinomas, whereas in melanomas, up-regulation of CEACAM1 is accompanied by poor overall survival. Beyond its prognostic value in cancers, CEACAM1 has often been referred to as a marker for tissue differentiation, especially in the context of mammary gland differentiation and mammary epithelial lumen formation [37–39]. Shively's group has demonstrated that CEACAM1-S expression was mandatory to establish acinar lumen formation *in vitro* [40–42]. In addition, CEACAM1 expression was also shown to revert malignant mammary cells to a differentiated, lumen-forming phenotype *in vitro* [41]. Intriguingly, they identified a direct molecular interaction between the CEACAM1-L cytoplasmic domain and β-catenin *in vitro*. However, *in vivo* evidence to corroborate these data and to connect CEACAM1-L and Wnt signaling in breast cancer development is lacking so far.

Based on these observations, we hypothesized that CEACAM1-L could negatively modulate the Wnt/β-catenin signaling by retaining β-catenin at the cell membrane, analogous to the role of E-cadherin (CDH1) *in vitro* [38]. Activation of the canonical Wnt signaling pathway involves re-localization of β-catenin from the cell membrane to the nucleus, where it initiates the transcriptional program that induces EMT [43]. The present study reveals that CEACAM1-L expression reduces β-catenin phosphorylation at positions Y86, a post-translational modification known to sustain activity of the Wnt-pathway [44, 45]. Our data strongly support a CEACAM1-dependent repression of β-catenin-phosphorylation at Y86 based on recruitment of SHP- 2. We furthermore observed that CEACAM1-L not only serves as a membrane scaffold for β-catenin and SHP-2, but also promotes Wnt-pathway inhibitory phosphorylation at S33/S37/T41 [46]. Loss of CEACAM1 in WAP-T tumor cells produced increased canonical Wnt signaling and promoted cellular invasiveness *in vitro*, and importantly, dramatically enhanced the metastasis rate of mammary adenocarcinomas in CEACAM1^null^ mice *in vivo*. Also, analyses of a publicly available database revealed that CEACAM1 expression is inversely correlated with survival of human mammary carcinoma patients.

Together, these results suggest that CEACAM1 acts as a gatekeeper for the maintenance of the epithelial phenotype in breast cancer cells *in vitro* and *in vivo*.

## RESULTS

### Mesenchymal markers are specifically up- regulated in CEACAM1^low/−^ G-2 cells

**Figure 1:**
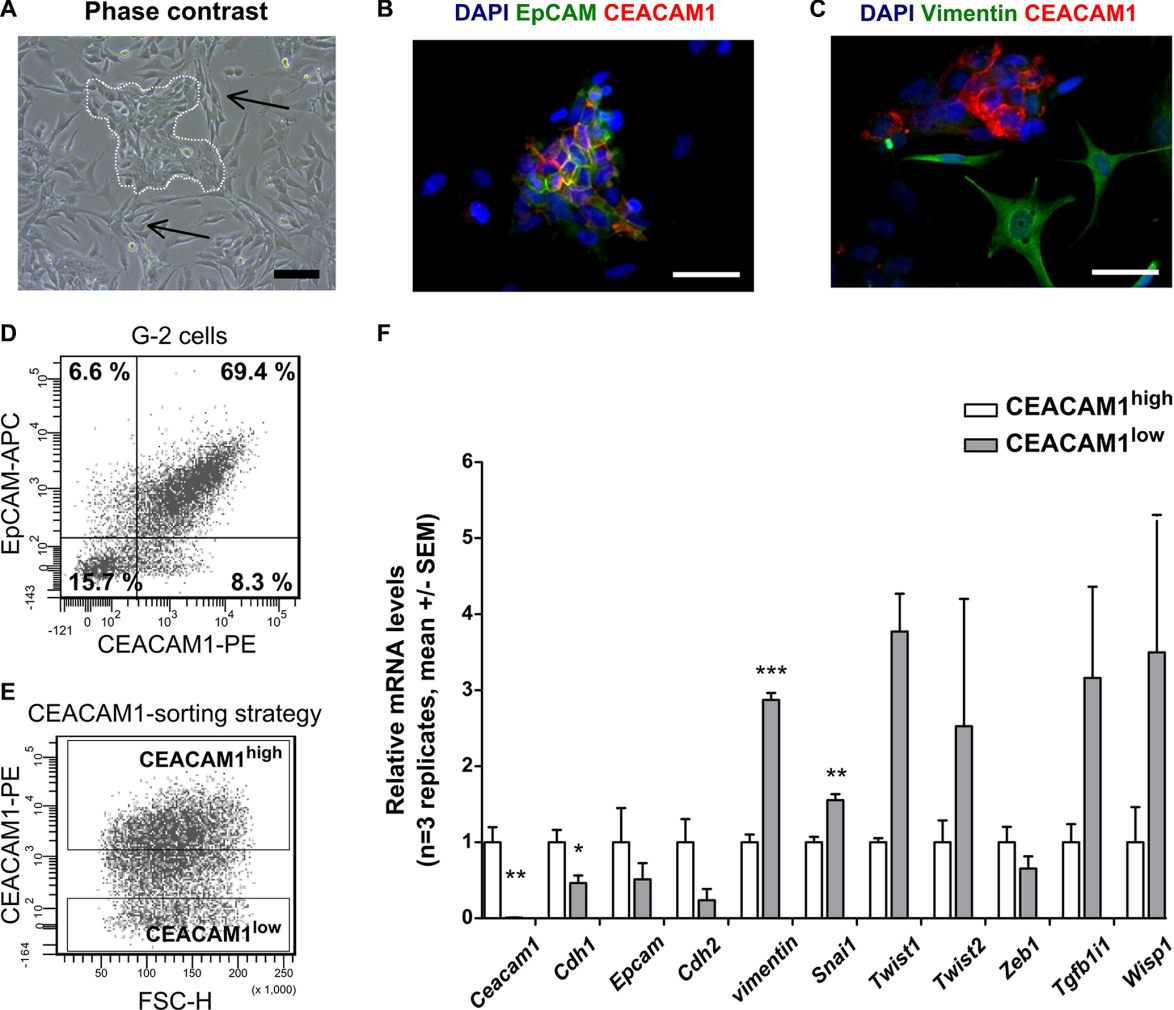
CEACAM1-expression and epithelial and mesenchymal properties of G-2 cells (**A–C**) microscopic images depict the epithelial and mesenchymal morphology and marker expression in phase contrast (A) and fluorescence microscopy (B, C). In (A), the epithelial subpopulation of the G-2 cells exhibits a cobble-stone morphology in distinct cell colonies (white dotted line), whereas the mesenchymal fraction of the cell population (black arrows) exhibits extended, spindle-shaped cell bodies. (B, C) CEACAM1 expression (red) is shown in the epithelial cells, where it is co-localized with EpCAM (green) at the cellular membrane (B). In contrast, mesenchymal, Vimentin-expressing cells (C, green) do not express CEACAM1. Flow cytometric analyses for EpCAM and CEACAM1 expression (**D, E**) and sorting strategy for CEACAM1^high^ and CEACAM1^low^ G-2 cells (E) are shown. (**F**) the relative expression levels of characteristic epithelial and mesenchymal markers genes are compared after cell sorting according to (E) between CEACAM1^high^and CEACAM1^low^ G-2 cells (*Ceacam1*, *Cdh1*, *Epcam*, *Cdh2*, *vimentin*, *Snai1*, *Twist1*, *Twist2*, *Zeb1*, *Tgfb1i1*, *Wisp1*). Expression data from CEACAM1^high^ and CEACAM1^low^ cells are shown in white and grey bars, respectively. Data shown here are representative of three independently repeated experiments in three replicates. Statistical analyses: Student's *t*-test. Scale bars: A: 100 μm, B and C: 50 μm.

In this study, we characterize CEACAM1-dependent effects on EMT *in vitro* and *in vivo*. For our *in vitro* studies, we used G-2 cells derived from primary mammary adenocarcinomas grown in WAP-T mice [12]. G-2 cells exhibit cancer stem cell-like properties and are composed of mixed epithelial and mesenchymal subpopulations *in vitro*, as shown in the phase contrast image in Figure 1A. These two cellular subsets are complementary and reciprocally switch between an epithelial-like (white dotted line) and a mesenchymal-like phenotype (black arrows), or vice versa [12]. As demonstrated by immune fluorescence (Figure 1B, 1C), the epithelial-like subpopulation co-expresses the epithelial markers CEACAM1 (red) and EpCAM (green) at the membrane (Figure 1B), whereas the mesenchymal-type cells are negative for CEACAM1 or EpCAM, but prominently express the mesenchymal marker Vimentin (green in Figure 1C), (Figure 1C). These observations suggest that an epithelial-like phenotype is connected to expression of CEACAM1.

To further identify regulatory pathways that determine epithelial cell differentiation in a CEACAM1-dependent manner, we sorted CEACAM1^high^ and CEACAM1^low^ G-2 cells using fluorescence activated cell sorting (FACS) and analyzed their individual epithelial and mesenchymal gene signatures (Figure 1D–1F). As shown in Figure 1D, the majority of the G-2 cells co-express CEACAM1 and EpCAM at variable levels. Previous experiments demonstrated that abrogation of CEACAM1 expression in G-2 cells greatly interferes with their survival (F. Wegwitz, A.K. Horst, W. Deppert, unpublished observations). Therefore we compared enriched CEACAM1^high^ with CEACAM1^low^ G-2 populations (Figure 1E) with regards to their expression of EMT marker genes as in Lenfert et al. (Figure 1F) [18] We found that CEACAM1^high^ cells expressed significantly higher RNA amounts of *E-cadherin (Cdh1)*, and significantly lower RNA amounts of the EMT-drivers *snail homolog 1* (*Snai1)* and *vimentin* compared to CEACAM1^low^ G-2 cells (Figure 1F). In addition, up-regulation of the mesenchymal marker genes *Twist-related protein 1 and 2* (*Twist1*, *Twist2*), *Transforming growth factor beta 1-induced transcript 1 (Tgfb1i1)* and *Wnt-inducible signaling pathway protein 1 (Wisp1)* was detected in CEACAM1^low^ G-2 cells (Figure 1F).

### CEACAM1 co-localizes and co-precipitates with β-catenin in murine G-2 cells

**Figure 2:**
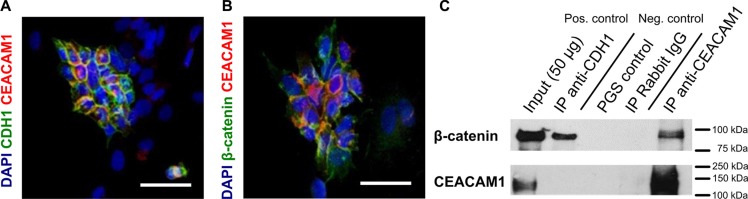
Co-localization and physical interaction of CEACAM1 with β-catenin (**A** and **B**) co-localization of E-cadherin (green, A) or β-catenin (green, B) and CEACAM1 (red) is shown in the epithelial subpopulation of G-2 cells in immune fluorescence microscopy. (**C**) results from co-precipitation of CEACAM1 with β-catenin or CDH1 from 200 μg G-2 cell lysates are shown, followed by immunoblotting with anti β-catenin (upper panel) and anti-CEACAM1-antibodies (lower panel). Polyclonal anti-rabbit immunoglobulins or protein A/G sepharose (PAS/PGS) were used as controls. β-catenin co-precipitates with CEACAM1 and E-cadherin, whereas precipitation of E-cadherin does not reveal binding of CEACAM1. Scale bars: 50 μm.

To ascertain if our hypothesis that CEACAM1 functions as a component of the EMT switch, we next analyzed whether E-cadherin, β-catenin and CEACAM1 interacted at the protein level. The interaction of human CEACAM1 with β-catenin has been demonstrated before *in vitro*: human CEACAM1-L contain a β-catenin binding motif in its cytoplasmic tail [38, 47]. This is missing in CEACAM1-S, but its cytoplasmic domain binds to Actin and protein kinase C. In Jurkat cells, CEACAM1-dependent regulation of Fas-mediated apoptosis depends on its interaction with β-catenin [48]. Similarly, in a model of colonic carcinogenesis (APC^1638N/+^:*Ceacam1* ^−/−^mice), a correlation was found between the absence of CEACAM1 and β-catenin nuclear translocation [38, 49]. To verify the putative interaction between CEACAM1 and β-catenin in G-2 cells, we performed co-localization studies and co-immunoprecipitation with β-catenin. As shown in Figures 2A and 2B, CEACAM1-expressing cells exhibit a prominent membrane co-labeling of CEACAM1 and E-cadherin, or of CEACAM1 and β-catenin, respectively. Furthermore, we show in co-immunoprecipitation assays that CEACAM1 physically interacts with β-catenin in G-2 cells, whereas E-cadherin only showed binding with β-catenin (Figure 2C). These observations support our hypothesis that distinct ß-catenin fractions are maintained at the cell membrane by either interacting with E-cadherin or CEACAM1.

### Reduction of CEACAM1 expression facilitates EMT and Wnt signaling

**Figure 3:**
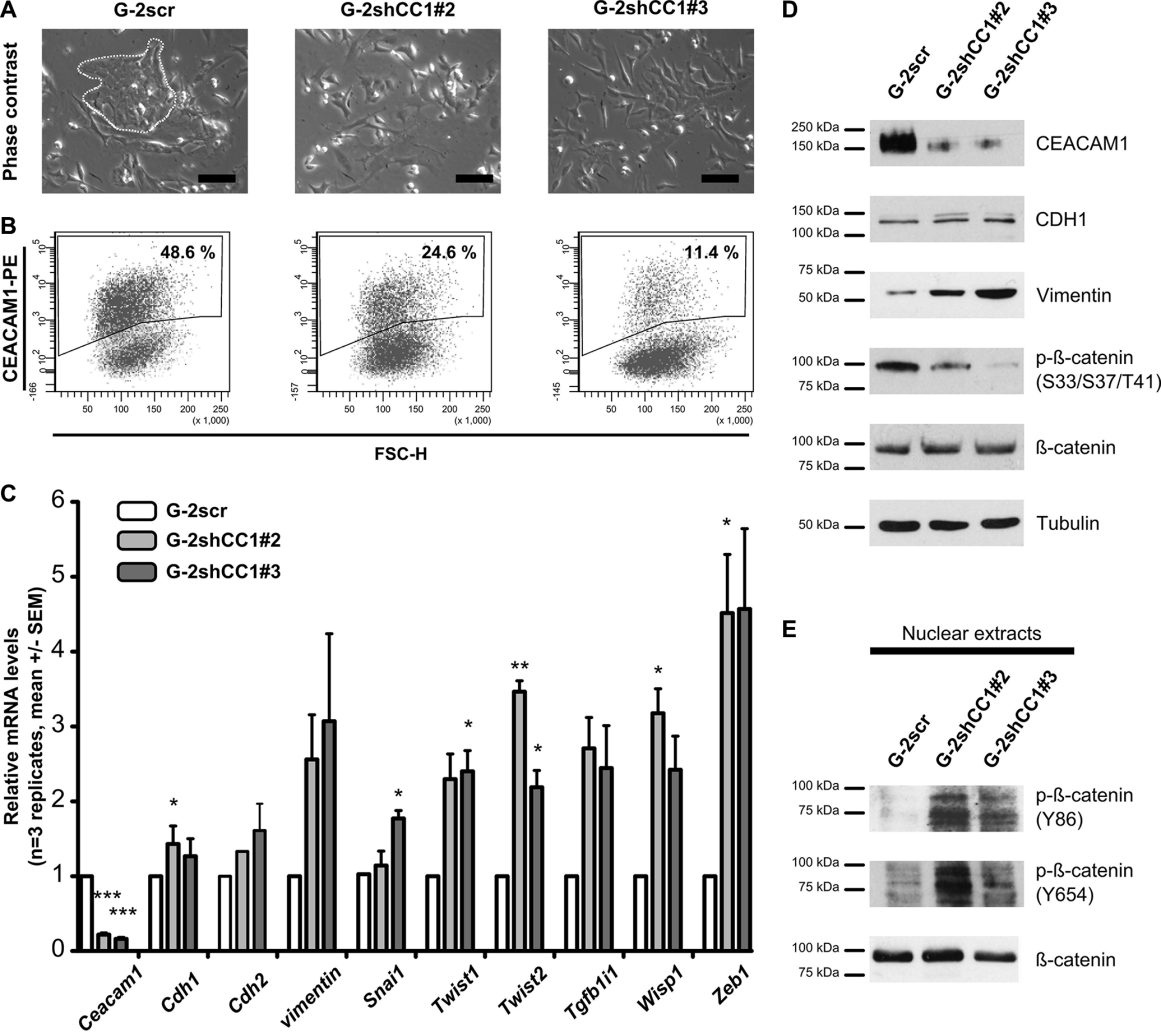
Analyses of EMT after down-regulation of CEACAM1 expression in G-2 cells (**A**) CEACAM1 expression was down-regulated in stable anti-CEACAM1 shRNA –transfectants (clones G-2shCC1#2 and G-2shCC1#3); as a control, a cell clone stably expressing scrambled shRNAs was used (G-2scr). Whereas the epithelial phenotype is preserved in G-2scr cell colonies (left panel, white dotted line), the G-2shCC1#2 and G-2shCC1#3 populations exhibit a rather mesenchymal phenotype, also evident by poor intercellular adhesion and scattered cell growth (A, middle and right panel). Scale bars: 100 μm. (**B**) Dot plot histograms from flow cytometry analyses of the different G-2 populations are shown; they reveal maintenance of CEACAM1 expression (48.6%) in the G-2scr cells, and reduction of the fraction of CEACAM1-positive/CEACAM1^high^ cells to 24.6% and 11.4% in the G-2shCC1#2 and G-2shCC1#3 clonal populations. (**C**) Comparative qRT-PCR expression analyses of key epithelial and mesenchymal marker genes (*Ceacam1*, *Cdh1*, *Epcam*, *Cdh2*, *vimentin*, *Snai1*, *Twist1*, *Twist2*, *Zeb1*, *Tgfb1i1*, *Wisp1*) in G-2scr, G-2shCC1#2 and G-2shCC1#3 cells. Data from G-2scr cells, G-2shCC1#2 and G-2shCC1#3 cells are shown in white, light grey and dark grey bars, respectively. Data sets are expressed as means ± SEM and were repeated three times independently in technical triplicates. Statistics: Student's *t*-test. (**D**) Comparative, representative Western blot analyses of CEACAM1, CDH1, Vimentin, Tubulin, and phosphorylated at S33/S37/T41 in β-catenin in G-2scr, G-2shCC1#2 and G-2shCC1#3 cells. (**E**) Western blots of nuclear extracts showing tyrosine phosphorylation (Y86, Y654) of β-catenin in G-2scr, G-2shCC1#2 and G-2shCC1#3 cells.

As subcellular β-catenin localization and its phosphorylation determine Wnt signaling activity, we subsequently investigated whether β-catenin phosphorylation and Wnt signaling were connected to CEACAM1-L expression and could influence EMT. We asked whether different levels of CEACAM1 could modulate the activation of Wnt/β-catenin signaling and thereby affect the balance between the epithelial and mesenchymal phenotypes in G-2 cells. We thus generated variants with different levels of CEACAM1 expression using shRNA technology (Figure 3A and 3B; G-2scr, G-2shCC1#2 and G-2shCC1#3). With approximatively 50% CEACAM1^+^ cells, the cell line obtained after transduction with scrambled shRNA construct (G-2scr) closely resembled the G-2 parental cell line (Wegwitz, F., Deppert, W., unpublished observations and Figure 1). Furthermore, two stable cell lines with clear reduction in their CEACAM1^+^ fractions (24% and 11%, respectively) were selected (Figure 3B; middle and right panel). Interestingly, gradual reduction of CEACAM1 expression enlarged the proportion of mesenchymal-like cells (Figure 3A): epithelial-like cells are decreased in the G-2shCC1#2 population, and are no longer detectable in the G-2shCC1#3 population (Figure 3A, right upper panel). This indicates that a minimal proportion of CEACAM1-expressing cells, or minimal CEACAM1 levels are required for maintenance of the epithelial phenotype. In addition, intercellular adhesion appeared markedly reduced in the two CEACAM1^low^ populations, where a cobblestone-like cellular morphology was largely absent (Figure 3A; G-2shCC1#2 and G-2shCC1#3), such that individual cells remained scattered and did not form epithelial-like colonies.

To document CEACAM1-dependent regulation of the epithelial or mesenchymal phenotype, we performed RT-qPCR and analyzed the expression of key regulators of EMT: we first validated the down-regulation of *Ceacam1* gene transcripts in the CEACAM1^low^ G-2shCC1#2 and G-2shCC1#3 cell lines (Figure 3C). Strikingly, we observed an up-regulation of *Tgfb1i1, Twist1, Twist2, vimentin, Wisp1* and *Zeb1*. Congruently, at the protein levels, this EMT-weighted signature is corroborated with reduction of CEACAM1 expression and increased concentration of Vimentin (Figure 3D). Interestingly, the levels of E-cadherin remained unaltered, pointing towards enhanced EMP rather than a complete EMT, where E-cadherin expression is frequently lost (Figure 3D). Furthermore, we demonstrate that increases in cellular plasticity in CEACAM1^low^ cells coincide with a reduction of β-catenin phosphorylation on S33/S37/T41. These serine and threonine residues are subject to phosphorylation by glycogen synthase kinase 3β, and target β-catenin for poly-ubiquitinylation and degradation, and thus negatively regulate its transcriptional activity [46, 50, 51]. Contrary to phosphorylation on S33/S37/T41, β-catenin tyrosine phosphorylation on Y86 is enhanced in CEACAM1^low^ cell populations exhibiting comparable total nuclear β-catenin levels (Figure 3E). In contrast to S33/S37/T41 phosphorylation, phosphorylation of this tyrosine residue stimulates the transcriptional activity of β-catenin [44, 45]. Taken together, these findings support our data that expression of CEACAM1 interferes with activation and nuclear translocation of β-catenin by inhibiting its phosphorylation at Y86.

### CEACAM1 suppresses β-catenin transcriptional activity

**Figure 4:**
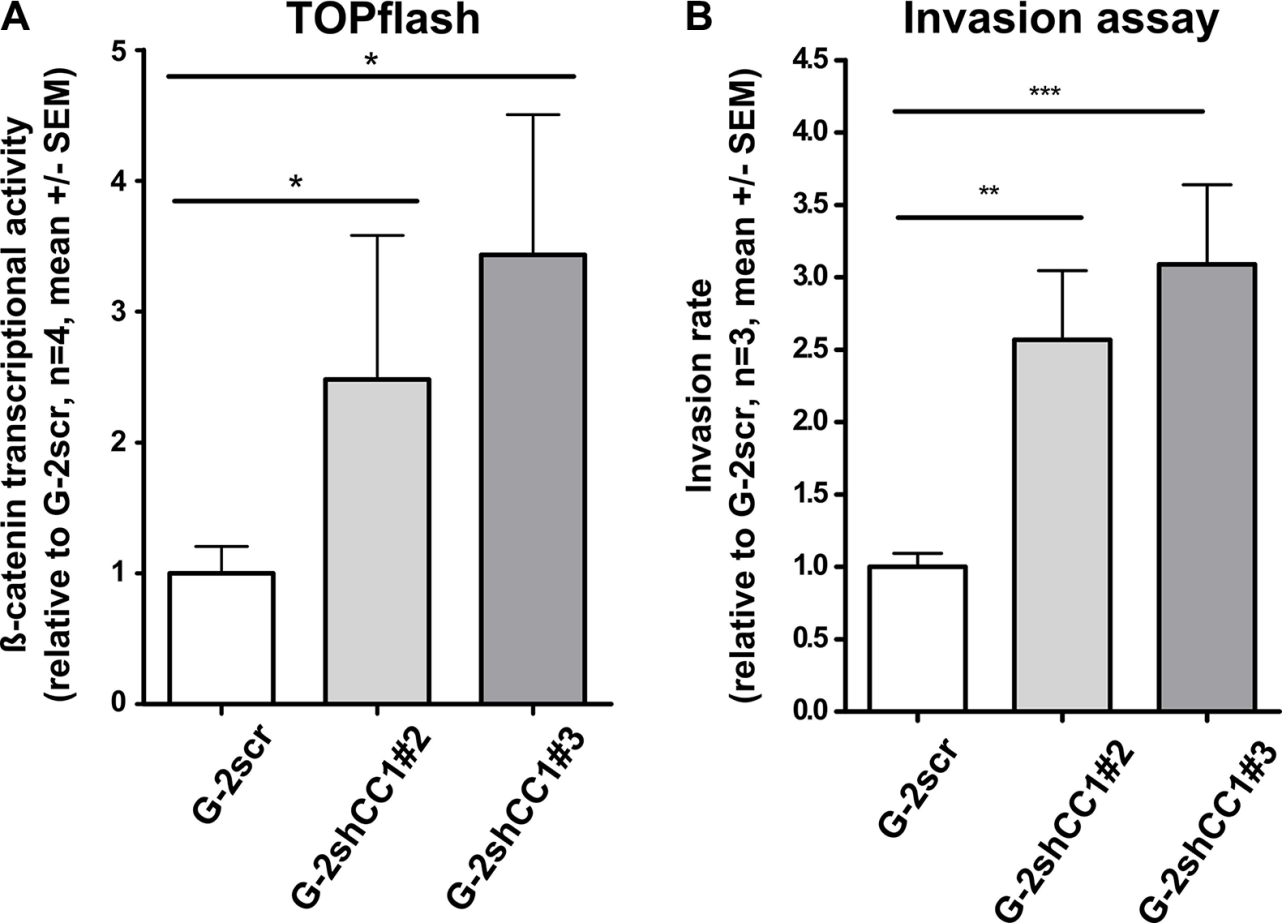
Comparison of β-catenin luciferase reporter activity and invasion potential of G-2 cells with normal or reduced CEACAM1 expression (**A**) Relative activity of the β-catenin-inducible promoter is compared in Luciferase assays (TOPflash assay: β-catenin-inducible promoter with intact TCF/LEF binding sites) in G-2 cells with normal (G-2scr) or reduced (G-2shCC1#2 and G-2shCC1#3) CEACAM1 expression. As a control, G-2scr, G-2shCC1#2 and G-2shCC1#3 populations were transfected with a control plasmid containing mutant TCF/LEF binding sites in the β-catenin inducible promoter (TOPflash). TOPflash values were calibrated to FOPflash results and normalized to β-catenin-inducible promoter activity in G-2scr cells. Data sets are expressed as means ± SEM and were repeated three times in quadruplet analyses. (**B**) Comparison of invasive capacities of G-2scr, and G-2shCC1#2 and G-2shCC1#3 cells in a basal membrane (BME) invasion assay; data from G-2scr, and G-2shCC1#2 and G-2shCC1#3 cells are shown in white, light grey and dark grey bars, respectively. Data sets are expressed as means ± SEM and were repeated three times.

To further corroborate our findings on the crosstalk between CEACAM1 and Wnt signaling, we performed luciferase-based β-catenin activity reporter assays using T-cell factor/lymphoid enhancer factor (TCF/LEF) transcription factor promoter constructs (TOP-FLASH); as a control, constructs harboring mutated TCF/LEF-binding site were used (FOP-FLASH). As previously reported in colon cancer cells [49], we found that reduction of CEACAM1 expression induced TCF/LEF dependent β-catenin transcriptional activity (Figure 4A). In agreement with our data presented above, functional investigations demonstrated that Matrigel^TM^ cellular invasion was enhanced by approximately 2–3 -fold in cell clones with reduced CEACAM1 expression and increased β-catenin–inducible promoter activity (Figure 4B). These observations support our hypothesis that CEACAM1 expression blocks mesenchymal conversion of G-2 cells and thus impairs cellular motility.

### CEACAM1 overexpression in G-2 cells inhibits EMT-related gene expression

**Figure 5:**
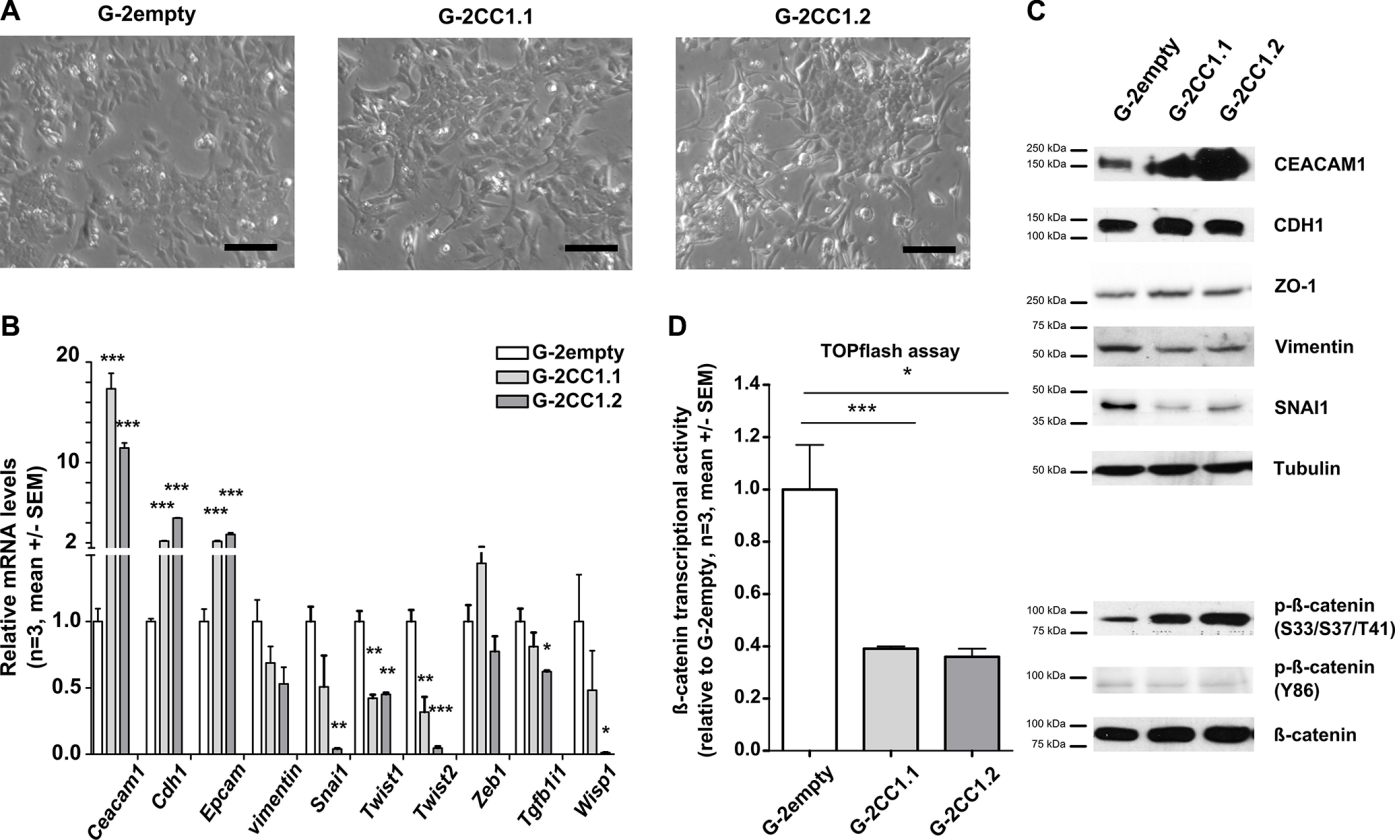
Overexpression of CEACAM1 in G-2 cells reduces the EMT phenotype (**A**) Phase contrast microscopic images document maintenance of the epithelial phenotype in G-2 cells, as well as in G-2 cells overexpressing CEACAM1 (G-2CC1.1, middle and G-2CC1.2, right panel) Scale bars: 75 μm (**B**) Expression analyses of key epithelial and mesenchymal marker genes (*Ceacam1*, *Cdh1*, *Epcam*, *Cdh2*, *vimentin*, *Snai1*, *Twist1*, *Twist2*, *Zeb1*, *Tgfb1i1*, *Wisp1*) in G-2empty, G-2CC1.1 and G-2CC1.2 populations via qRT- PCR. Data from G-2empty, G-2CC1.1 and G-2CC1.2 cells are shown in white, light grey and dark grey bars, respectively. Data sets are expressed as means ± SEM and were repeated three times in triplicate analyses. (**C**) Western blot for proteins levels comparison of CEACAM1, CDH1, ZO-1, Vimentin, SNAI1, β-catenin and phosphorylated β-catenin; (S33/S37/T41 and Y86). Tubulin levels were used as loading controls. (**D**) Relative levels of β-catenin-inducible promoter activity assessed by TOPflash-Luciferase assays in G-2empty, G-2CC1.1 and G-2CC1.2 cells. TOPflash values were calibrated to FOPflash results and normalized to β-catenin-inducible promoter activity in G-2scr cells. Data sets are expressed as means ± SEM and were repeated three times in triplicate analyses.

Our data above provide further evidence that EMT is facilitated when CEACAM1 expression is reduced. However, whether re-introduction or over-expression of CEACAM1 in epithelial cells inhibits EMT has not been studied so far. Therefore, we overexpressed CEACAM1 in G-2 cells and analyzed changes in their cellular morphology as well as the transcriptional activity and protein levels of EMT-related genes (Figure 5). We also assayed β-catenin transcriptional activity and its phosphorylation status in response to CEACAM1 overexpression. Phase contrast images depict that enhanced CEACAM1 expression imposes an epithelial-like phenotype on G-2 cells (Figure 5A). This clear change in cellular morphology is accompanied by a congruent change in the expression of *E-cadherin*, *Epcam* and *Tgfb1i1*, whereas the majority of EMT inducers, *Wisp1*, *Twist1, Twist2 and Snai1* were down-regulated significantly (Figure 5B). Changes in expression of *vimentin* is only weak on RNA levels (Figure 5B), but protein levels of SNAI1 and Vimentin were significantly reduced in G-2 cells overexpressing CEACAM1 (Figure 5C). In addition, S33/S37/T41 phosphorylated forms of β-catenin were increased after enforced CEACAM1 expression (Figure 5C). In contrast, protein levels of E-cadherin and those of ZO-1, a gatekeeper of epithelial polarity, were only moderately increased, whereas Y86 phosphorylation was slightly decreased (Figure 5C). In line with these findings, transcriptional activity of β–catenin inversely correlated with CEACAM1 expression in G-2 cells (Figure 5D). The reduction of β–catenin transcriptional activity was even more pronounced when canonical Wnt signaling was activated by stimulation with WNT3a in CEACAM1 overexpressing G-2 cells (Supplementary Figure S1A).

### SHP-2 binds to CEACAM1 and maintains the epithelial phenotype in G-2 cells

**Figure 6:**
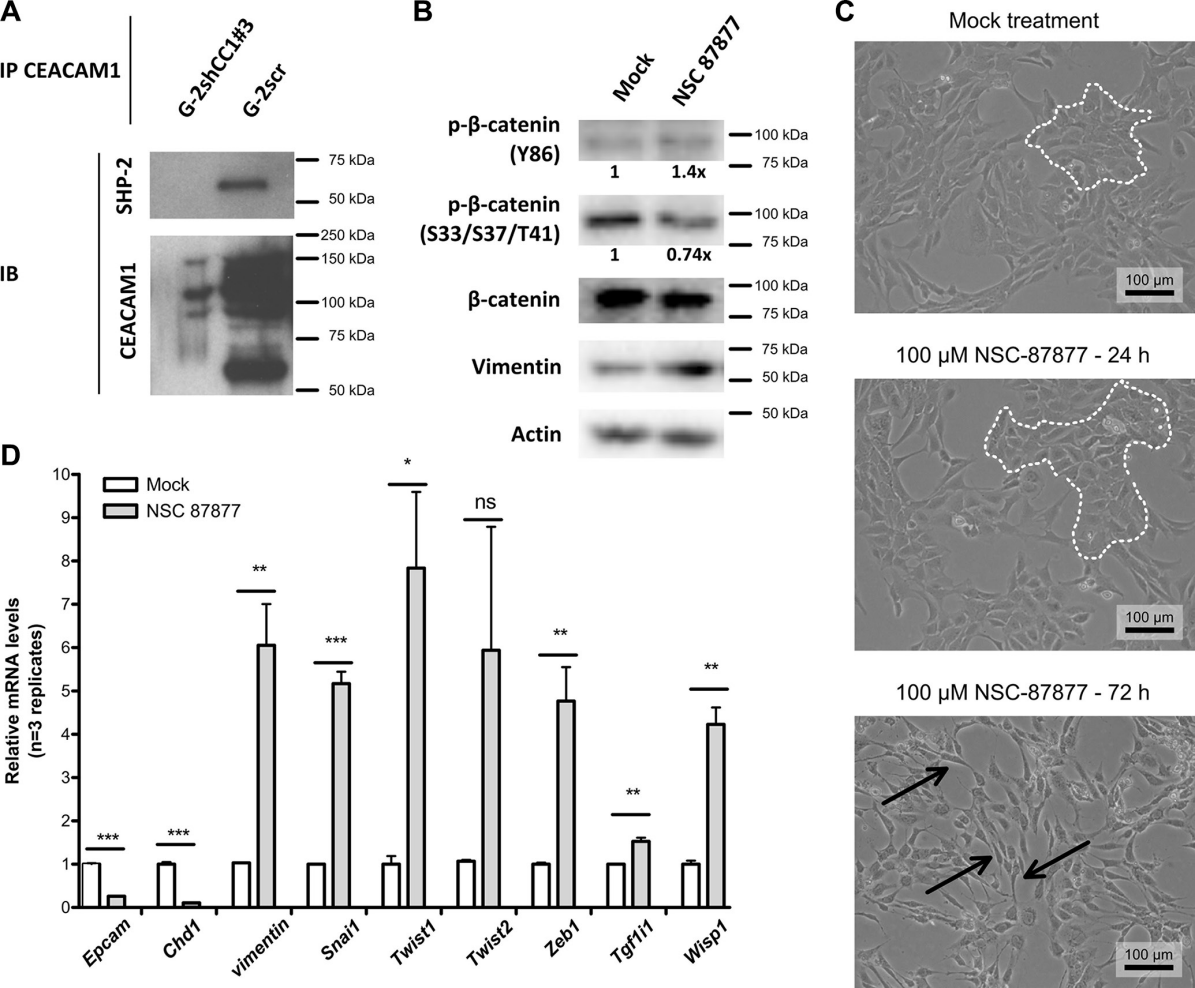
SHP-2 interacts with CEACAM1 and blocks EMT in G-2 cells (**A**) Co-immunoprecipitation of SHP-2 with CEACAM1 in CEACAM1-expressing G-2scr cells, but not in G-2shCC1#3 with reduced CEACAM1 levels, demonstrates a physical interaction between SHP-2 and CEACAM1. (**B**) assessment of β-catenin phosphorylation by Western blot in cells without (mock) or with SHP-2 inhibitor treatment (NSC87877, 100 μM, 24 hrs): treated cells display moderately increased levels of β-catenin phosphorylation at Y86 as well as reduced β-catenin phosphorylation at S33/S37/T41; concomitantly, the expression level of Vimentin is increased; quantities were normalized relative to β-catenin levels. (**C**) Phase contrast images of CEACAM1-expressing G-2 cells treated with 100 μM SHP- 2 inhibitor NSC-87877 for 24 hrs or 72 hrs shows that mesenchymal transformation of the G-2 cells increases with time in comparison to mock treated cells; epithelial cell clusters are indicated by white dotted lines, mesenchymal cells with poor intercellular contacts and elongated cell bodies are indicated by black arrows; scale bar: 100 μm. (**D**) RT-qPCR-based gene expression analyses of EMT-signature marker genes in G-2 cells with (shaded bars) or without (white bars) SHP-2 inhibitor treatment (NSC-87877, 100 μM, 72 hrs). Note that the expression of *Epcam* and *E-cadherin* is significantly reduced cells with SHP-2 inhibition, whereas EMT drivers such as *vimentin*, *Snai1*, *Twist1*, *Zeb1*, *Tgf1i1*, and *Wisp1* are elevated; *Twist2* did not exhibit significant alteration of expression.

Since endothelial SHP-2 is known to regulate the recovery of adherent junctions through control of β-catenin phosphorylation, we tested whether SHP-2 and CEACAM1 could interact in CEACAM1-expressing G-2 cells [52]. Indeed, we were able to confirm a physical interaction of CEACAM1 and SHP-2 in pervanadate-treated G-2 cells (Figure 6A), but not in G-2 cells with reduced CEACAM1-levels (G-2shCC1#3). NSC-87877 has been described as small molecule specifically inhibiting phosphatase activity of SHP-2 as well as of SHP-1 [53]. Since SHP-1 protein levels were not detectable in G-2 cells (data not shown), we assumed that the effects of NSC-87877 pharmacological inhibition are specific to SHP-2 inhibition. To test whether SHP-2 activity was required to maintain the epithelial phenotype of G-2 cells, we treated G-2 cells with NSC-87877 (Figure 6B, 6C) and compared β-catenin phosphorylation (Figure 6B), cellular morphology (Figure 6C), and the epithelial and mesenchymal marker signatures (Figure 6D). We found that SHP-2 inhibition produced a mild increase in β-catenin Y86 phosphorylation (1.4× compared to control) after 24 hours, whereas phosphorylation of β-catenin inhibitory residues S33/S37/T41 was reduced (approximatively 0.74×) (Figure 6B). Accordingly, we observed increased Vimentin expression in G-2 cells treated with the SHP1/2 inhibitor (Figure 6B). Congruently, the proportion of mesenchymal-like cells increased over time under NSC-87877 treatment (Figure 6C; epithelial cell colonies are marked by white dotted lines, mesenchymal cells are indicated by black arrows). The importance of intact SHP-2 signaling for the maintenance of an epithelial phenotype in G-2 cells was corroborated by analyses of the gene expression profiles of EMT inducers (Figure 6D). We found that the expression of mesenchymal signature genes was strongly enhanced upon SHP-2 inhibition in G-2 cells. The majority of EMT markers and regulators, such as *Vimentin*, *Snai1*, *Twist1*, *Zeb1*, *Tgf1i1* and *Wisp1*, were significantly upregulated after NSC-87877 treatment for 72 hrs, accompanied by significant down-regulation of epithelial marker genes, such as *Epcam* and *E-cadherin* (Figure 6D). Here, we demonstrate that CEACAM1 is critical for the maintenance of the epithelial phenotype in G-2 cells by regulating β-catenin activity through SHP-2-dependent de-phosphorylation of Y86, accompanied by increased phosphorylation on residues S33/S37/T41.

### Enhanced activation of Wnt signaling and increased pulmonary metastasis in WAP-T/CEACAM1^null^ mice

**Figure 7:**
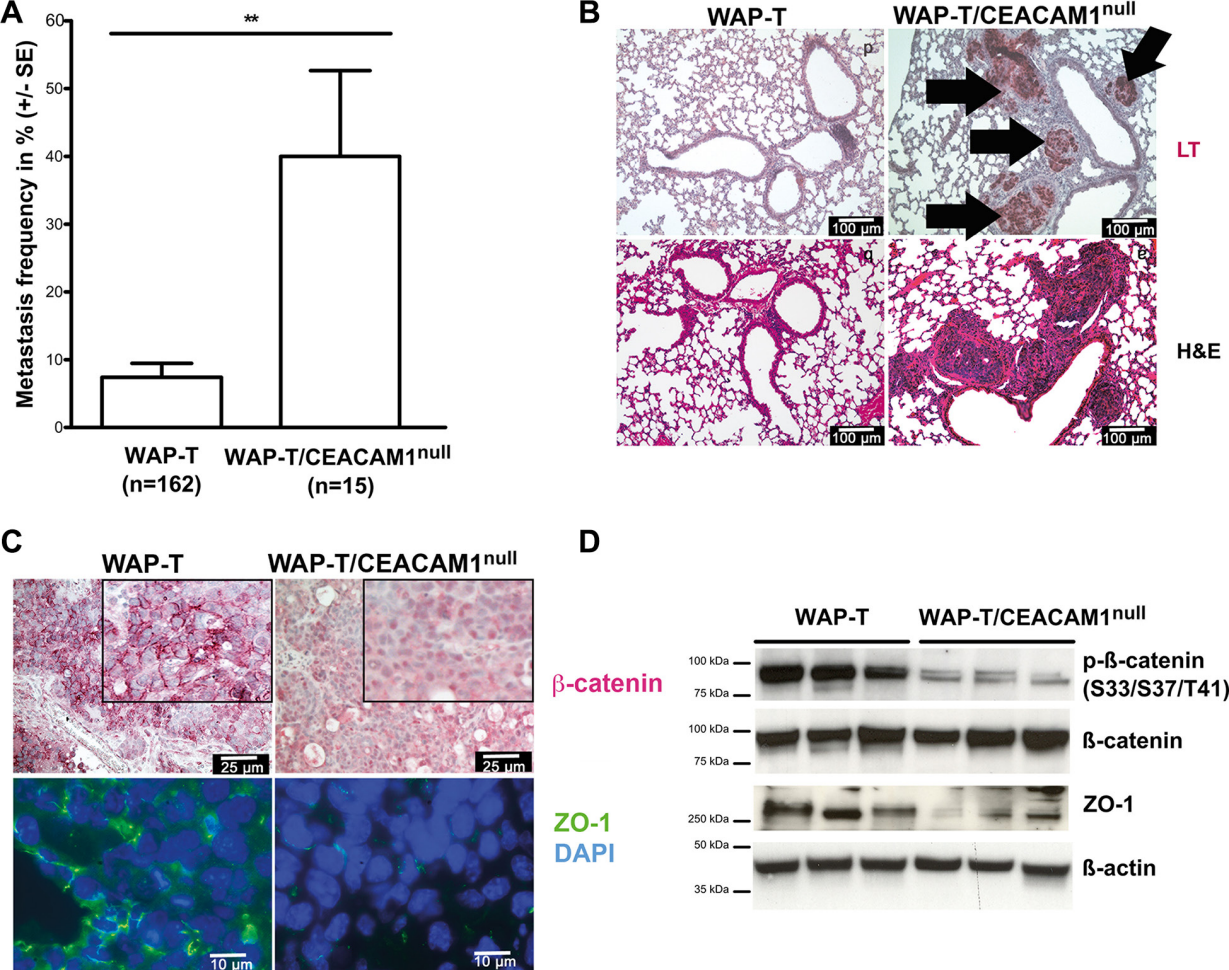
Loss of CEACAM1 *in vivo* enhances EMT and pulmonary metastasis of murine mammary adenocarcinomas (**A**) Quantitative evaluation of pulmonary metastasis in WAP-T and WAP-T/CEACAM1^null^ mice presents significant differences in metastastic frequencies. Data shown in percent ± standard error (**B**) Histological analyses of metastasis-free lung tissue and pulmonary metastases in WAP-T and WAP-T/CEACAM1^null^ mice reveal LT-expressing metastases in WAP-T/CEACAM1^null^ mice (upper panel, bold arrows point at clusters of intra-vascular and inter-septal metastases). These were identified in H&E stained tissue sections of tumor-bearing WAP-T mouse (lower panels), whereas no metastases were found in the depicted tissue sections of a tumor-bearing WAP-T mouse; (**C**) Immune histological analyses of expression and localization of β-catenin and ZO-1 in mammary tumors from WAP-T and WAP-T/CEACAM1^null^ mice reveal differential localization of β-catenin (red staining) at cell membranes (WAP-T mice, inset enlarged) and in the nuclei (WAP-T/CEACAM1^null^ mice; insets enlarged). In immune fluorescence, ZO-1 (shown in green) is localized at cellular junctions in tumors from the WAP-T mice, but is largely absent from CEACAM1^null^ tumors. Nuclei were stained with DAPI (blue); scale bars are indicated (**D**) Analyses of protein expression levels of phosphorylated β-catenin (upper lane), and total β-catenin (second lane), as well as total ZO-1 protein in tumor lysates from WAP-T and WAP-T/CEACAM1^null^ mice. Protein levels were normalized to β-Actin (lower lane). Note that phosphorylation of β-catenin is clearly reduced in tumors from WAP-T/CEACAM1^null^ mice. Similarly, ZO-1 protein expression is reduced in WAP-T/CEACAM1^null^ mice. Scale bars as indicated.

To translate these findings into an *in vivo* model, we crossbred WAP-T mice with CEACAM1-deficient mice and analyzed the spontaneously grown mammary tumors and their progression and metastasis [10, 54]. As shown in Figure 7A, WAP-T/CEACAM1^null^ mice exhibit significantly higher rates of metastasis; in WAP-T mice, pulmonary metastases were found in approximately 8% of all mice examined. By contrast, pulmonary metastases were detected in approximately 40% of the tumor-bearing WAP-T/CEACAM1^null^ mice (Figure 7A, 7B). The position of the tumor-bearing mammary gland did not affect metastasis incidence (data not shown). Surprisingly, in H&E staining, morphology of primary lesions was similar in CEACAM1 wild-type and deficient mice ([54] and data not shown). When pulmonary metastases were analyzed for large T-Antigen (LT) expression, we found that in the majority of the mice examined, metastases were LT-positive (Figure 7B, upper panel). In the lower panel, intra-arterial and inter-septic pulmonary metastases are depicted.

In immunostainings for β-catenin and Zonula occludens protein 1 (ZO-1) expression, we found that β-catenin exhibited a distinct staining pattern at the cell membrane in WAP-T tumors (Figure 7C, upper panel). In contrast, β-catenin had preferentially translocated in the tumor cell nuclei in tumors from WAP-T/CEACAM1^null^ mice (Figure 7C, upper right panel). This observation corroborates our data that Wnt signaling is persistently activated in the absence of CEACAM1 (cf. Figures 3D, 3C, 3D and 4A). Furthermore, intercellular junctions were reduced in CEACAM1-negative tumors, as evidenced by reduction and focal loss of ZO-1 staining along cell membranes (Figure 7C, lower right panel). These data were confirmed in immunoblots, where a clear reduction of β-catenin phosphorylation at S33/S37/T41, and total reduction of ZO-1 levels were observed (Figure 7D).

Since loss of CEACAM1 represents a clear adverse prognosis parameter in WAP-T mouse mammary carcinomas, we took advantage of a publicly available database, www.klplot.com, to assess the impact of CEACAM1 expression levels on breast cancer patient survival [55, 56]. In line with our experimental results, CEACAM1 expression levels were inversely correlated with 10-yr survival rates of patients (Supplementary Figure S1B), irrespective of the particular tumor subtype (Supplementary Figure S1C–S1F).

In summary, our study demonstrates for the first time that loss of CEACAM1 expression facilitates EMT in murine mammary carcinoma *in vitro* and *in vivo*. Loss of CEACAM1 expression impairs negative modulation of Wnt signaling. Consequently, low CEACAM1 expression in breast cancer patients correlates with poorer outcome than high CEACAM1 expression.

## DISCUSSION

Dichotomous roles have been assigned to CEACAM1 in tumor development and progression: on the one hand, its expression has been correlated to increased tumor invasion in melanoma, lung carcinoma, in carcinomas of the thyroid, liver and colon; in these specimens, CEACAM1 is up- regulated during progression of the disease and has been established as an independent prognostic marker [57, 58]. On the other hand, in early stages, CEACAM1-L is down-regulated in tumor cells of different organs including neoplastic lesions of the colon, stomach, bladder and prostate [59–65]. Similarly, CEACAM1 is strongly expressed on normal breast epithelial cells and is down-regulated in benign as well as malignant breast cancer [31, 66–69]. As CEACAM1 can act as an inhibitor of tumor cell growth of several cancers, e.g. colon and prostate cancer, it was identified as a putative tumor suppressor [57, 58].

Based on our previous findings that overexpression of mutant p53 increases mammary carcinoma cell aggressiveness and is associated with an EMT gene expression signature and reduction of CEACAM1-expression, we analyzed the specific molecular role of CEACAM1 in EMT and cancer progression [11, 18]. In the present report, we demonstrate that CEACAM1 is a critical determinant in the regulation of the EMT switch. Taking advantage of the WAP-T mammary carcinoma mouse model and of the G-2 cancer stem cell line derived from it, we demonstrate for the first time that expression of CEACAM1-L can control the tumor cell aggressiveness *in vitro* by reducing EMT gene expression and invasion capabilities, and *in vivo* by decreasing its metastatic propensity. So far, only the CEACAM1-S isoform has been studied to greater detail with respect to mammary cell differentiation. However, the molecular basis for the interaction between β-catenin and CEACAM1-L has been determined *in vitro*, see below [38].

CEACAM1 is an integral component of adhesion junctions, where it co-localizes with E-cadherin, and β-catenin [70]. In culture systems, CEACAM1 is present on WAP-T cells with epithelial but not mesenchymal phenotype. It co-localizes and physically interacts with β-catenin, as previously reported for cancer cell lines [38, 49]. In normal epithelial cells, the majority of β-catenin is localized at the plasma membrane, in close apposition with adhesion molecules (e.g. E-cadherin) and thus is presumed to contribute to the maintenance of epithelial cell polarity. The structural integrity of the β-catenin/ E-cadherin complex and its associated cell junction properties is regulated by protein kinase and phosphatase activity [46]. The first evidence for an *in vivo* connection between CEACAM1 expression and the regulation of Wnt signaling were reported in the compound APC^1638N^:*Ceacam1* ^−/−^ mice. Here, absence of CEACAM1 in murine colonic adenocarcinomas led to enhanced nuclear translocation of β-catenin. Additionally, CEACAM1 expression was required in murine colonic tumor cells to down-modulate activity of the β-catenin-inducible promoter, which is in agreement with our findings [49]. Similarly, in the colonic tumor cell lines CT26 and CT51, interaction of β-catenin with CEACAM1-L, but not CEACAM1-S, was confirmed [49].

Furthermore, we provide a causal relationship between specific expression of CEACAM1-L and changes in phosphorylation of β-catenin on S33/37/T41. Reduction of CEACAM1 levels in cultured tumor cells and in endogenous tumors from WAP-T/CEACAM1^null^ mice was associated with decreased phosphorylation of β-catenin on S33/37/T41, a hallmark of activated Wnt/β-catenin pathway [51]. Phosphorylation of S33/37/T41 reduces β-catenin transcriptional activity by increasing affinity for E-cadherin and thereby its retention at the plasma membrane. On the other hand, unbound intracytoplasmic β-catenin is targeted for degradation by Skp, Cullin, F-box containing complex (SCF)-class E3-ubiquitin ligase [46, 71]. Importantly, this feature was associated with increased localization of β-catenin in the nucleus of the WAP-T tumor cells and consequently enhanced Wnt/β-catenin-pathway activity. Also, reduction of *Ceacam1* expression levels was coupled to a clear increase of tyrosine phosphorylation of β-catenin at Y86. Few reports exist on the functional consequences of distal β-catenin tyrosine phosphorylation. However, Coluccia et al. state that phosphorylation on Y86 enhances nuclear translocation and transcriptional activity [72]. In line, overexpression of CEACAM1-L leads to increased S33/37/T41 phosphorylation levels, reduced Y86 phosphorylation levels, and the inhibition of β-catenin transcriptional activity, suggesting that CEACAM1-L expression impacts on expression/activity of specific kinases and phosphatases responsible for these events.

Because of its potential tumor suppressive properties, research has largely focused on the CEACAM1 isoform with long cytoplasmic tail containing two immunoreceptor tyrosine-based inhibition motifs (ITIMs). These ITIMs are phosphorylated upon activation of kinases of the Src familiy as well as receptor tyrosine kinases like epidermal growth factor (EGF)- or Insulin-receptor (IR) [57, 73–75]. Consequently, they harbor binding motifs for inhibitory phosphatases containing Src homology 2 domains (SH2-domains), especially SHP-1 and SHP-2 [82]. Importantly, phosphatase activity of SHP-1 and SHP-2 down-modulates Src- or Syk-kinase signaling and reduces the activation of the mitogen-activated protein kinase (MAPK) cascade [20]. Reduction in the juxtamembrane SHP-1/−2 signaling has been described in *Ceacam1* ^−/−^ mice, where CEACAM1-dependent tyrosine phosphatase recruitment leads to persistent or exacerbated receptor activation. As a consequence, persistent toll-receptor (TLR)-signaling, hyperactivation of platelet adhesion, inadequate activation of IL-2R, EGFR and IR, as well as endothelial nitric oxide synthase or matrix-metalloproteinase 9 (MMP9) secretion were observed [27, 73, 76, 77].

The consequences of canonical Wnt/β-catenin signaling in the absence of CEACAM1 have not been fully elucidated. Simoneau et al. reported that ectopic expression of SHP-1 but not its inactive variant could inhibit phosphorylation of β-catenin on Y86 [78]. Similarly, Lee et al. described the ability of SHP-2 to dephosphorylate β-catenin at Y654 [79]. Considering the ability of both SHP-1 and SHP- 2 to dephosphorylate different tyrosine residues of β-catenin, thereby decreasing canonical Wnt-pathway activity, we describe here for the first time that the interaction with CEACAM1-L and SHP-2 contributes significantly to the regulation of the β-catenin phosphorylation status: SHP-2 inhibition moderately enhances phosphorylation of β-catenin on Y86, but this increase was even more pronounced if G-2 cells exhibited partial loss of CEACAM1 expression. Once SHP-2 activity is compromised by small molecule inhibitors, or CEACAM1 is downregulated in G-2 cells, phosphorylation of β-catenin on its inactivating S33/S37/T41 residues is impaired. This could be due to the fact that in G-2 cells, mesenchymal and epithelial populations overlap and that additional regulatory pathways funnel into the regulatory network that determines the dichotomous β-catenin phosphorylation pattern. Further studies will have to be conducted to reveal additional CEACAM1 interaction partners that participate in the CEACAM1-SHP-2-β-catenin signaling scaffold.

The present report clearly points towards an involvement of CEACAM1 in the regulation of the EMT-phenotype of tumor cells through Wnt/β-catenin pathway which impacts on cancer progression and metastasis. Even though the exact molecular mechanism remains to be deciphered, our data identify CEACAM1 as a gatekeeper in EMT that provides novel insights into the regulation of cancer invasion and metastasis.

## MATERIALS AND METHODS

### Animals

All animals were housed under SPF conditions in the animal facility of the University Medical Center Hamburg-Eppendorf and approved by Hamburg's Authority for Health and Environmental Protection (TVG 61/05; TVG 88/06). The model of prolactin-driven large T-Antigen (LT) expression in WAP-T mice (mice with prolactin-sensitive whey acidic protein-promoter driven expression of the large T-antigen) has been described [11, 54]. In brief, expression of the oncogenic LT is initiated during gestation, when lactation commences around gestational day 14. Following this induction, WAP-T mice undergo a sequence of dysplasia, DCIS, and adenocarcinoma formation within 8 months. Tumor growth, tumor histology, and metastasis were analyzed in both WAP-T and WAP-T/CEACAM1^null^ (*Ceacam1* ^−/−^) mice [54, 80].

### Immunohistology

For histological analysis tumor and lung specimens were processed as previously described [12, 54].

### Cell culture

The WAP-T cell line G-2 and derivatives were cultured in DMEM medium (PAA) supplemented with 10% FCS (PAA) in a humidified incubator at 37°C and 5% CO_2_. Generation of the G-2 cell line and its properties have been described previously [12]. Phase contrast pictures were obtained using a TS100 microscope (Nikon) equipped with a μEYE 1240ML-C-HQ camera (Imaging Development Systems).

### Plasmid construction and lentiviral transduction of G-2 cells (shCC1 and CC1cDNA)

For the ectopic expression of *Ceacam1-L* in G-2 cells, *Ceacam1* cDNA was extracted from the pcDNA-mCC1-4 via EcoRI-digestion and subsequently cloned into lentiviral vector LeGO-iG2 (using EcoRI restriction site), kindly provided by Dr. C. Stocking [81]. Sequences were confirmed by DNA sequencing (Seqlab, Germany). Production of lentiviral vectors and transduction of G-2 cells was performed as previously described [12]. Commercially available shRNA constructs against mouse *Ceacam1* mRNA using the pGIPZ vectorsystem were purchased at Open Biosystems (# RMM4532). The production of the lentiviral particles was performed according to the manufacturer instructions. GFP positivity was used to enrich cells that received the vector via fluorescence-activated cell-sorting.

### Flow cytometry

Flow cytometric analyses were performed on FACSCanto flow cytometer (BD Biosciences) and fluorescence-activated cell sorting was performed on a FACSAria cell sorter (BD Bioscience). Cells were stained as described [12] with a PE-labeled anti-CEACAM1 antibody (Biolegend, #134505) and/or an APC-labeled anti-EpCAM antibody (Biolegend, #118214). A complete list of primary antibodies is given in Supplementary Table S2.

### Quantitative real-time PCR

RNA from cultivated cells was purified using the innuPREP RNA Mini kit (Analytik Jena). Conversion of RNA into cDNA and quantitative real-time PCRs were performed as described [12]. *Hspa8* mRNA was used for normalization. Relative normalized transcript levels were calculated using the 2^−ΔΔCT^ algorithm. Quality and specificity of all PCR primers was validated by melting curve analyses and standard curve fitting. The primers used in this study are detailed in the Supplementary Methods section (Supplementary Table S1).

### Immunofluorescence staining

Immunofluorescence staining was performed as described previously [12] with the antibodies listed in Supplementary Table S2. Secondary antibodies used for immunofluorescence staining were appropriate DyLight^®^- or Alexa^®^Dye- conjugates obtained from Invitrogen/Molecular Probes or Dianova. Confocal microscopy was performed on a LSM 510 Meta (Zeiss). Conventional fluorescence microscopy was performed on a DMI6000B (Leica).

### Western blot analysis

Cells or tumors were lysed with Laemmli lysis buffer. Protein concentration was estimated using the BCA Protein Assay (Thermo Scientific). Equal amounts of protein were separated by SDS-PAGE and transferred onto nitrocellulose (Hybond, Amersham). The membrane was blocked with 5% skim milk in TBS-T for at least 60 min at RT. Primary antibodies were used as detailed in Supplementary Table S2. All secondary antibodies conjugated with horseradish peroxidase (Dianova) were used in dilutions of 1:5000–1:10000 in 5% skim milk (TBS-T). For the extraction of the nuclei, trypsinized cells were resuspended in cold DMEM + 10% FCS and centrifuged for 5 min at 300×g, 4°C. Cells pellets were washed with ice cold PBS, resuspended in Nuclei-Buffer I (10 mM Tris-HCl pH 7.9; 10 mM KCl; 15 mM MgCl_2_) and incubated for 10 min on ice. After centifugation (1000×g/ 2 min/4°C), the nuclei were lysed in Laemmli lysis buffer supplemented with 1 μl Benzonase (Roche) and incubated for 5 min at 95°C. Protein concentration was estimated using the BCA Protein Assay (Thermo Scientific). All buffers used here were supplemented with protease (cOmplete mini EDTA free, Roche) and phosphatase inhibitors (2 mM Na_3_VO_4_ and 5 mM NaF). For protein quantification, signal acquisition and measurement was performed on a Gel Doc XR+ System (BioRad).

### Immunoprecipitation

Cells were harvested in ice cold CHAPS buffer (100 mM Tris-HCl pH 7.4, 50 mM NaCl, 1 mM EDTA, 1% NP40, 0,1% CHAPS) supplemented 1 μl Benzonase as well as with protease and phosphatase inhibitors. Cell lysates were incubated on ice for 30 min and cell debris removed by centrifugation (12000×g/15 min/4°C). Protein concentration was determined by Bradford (Bio-Rad) assay; 200 μg of the protein lysates were used for immunoprecipitation. 10 μl protein G sepharose beads (GE Healthcare) were incubated at 4°C overnight with 2 μg anti-mouse CEACAM1 antibody CC1 (eBioscience) or anti-E-cadherin antibodies (Cell Signaling) as positive control. Precleaning was performed with 20 μl protein G sepharose at 4°C overnight. The precleaned lysates were then incubated with appropriate antibody-coupled protein G sepharose beads over night at 4°C. Co-precipitation of SHP-2 with CEACAM1 was performed after pervanadate treatment of G-2 cells using biotinylated anti-CEACAM1 antibodies (eBioscience), streptaviding sepharose (ThermoFisher) and anti-SHP-2 antibody (Cell Signaling Technology), as described in [82]. A complete list of primary antibodies is given in Supplementary Table S2.

### Invasion assay

Cells were harvested and kept for at least 3 hrs in serum-free DMEM. 2.5 × 10^4^ cells in medium without FCS were transferred onto basement membrane coated inserts (CultreCoat^®^ Medium BME Cell Invasion Assay, Trevigen). The lower chamber was filled with DMEM supplemented with 10% FCS as chemo-attractant, and the assay was incubated for 72 hrs at 37°C. Cells attached to the bottom of the inserts were fixed with 4% PFA and stained with DAPI. Cells were counted under a fluorescence microscope (Leica DMLB) at 10× magnification. Experiments were performed three times in triplicates.

### Luciferase assay

1 × 10^5^ cells in 12-wells were transfected with Renilla Luciferase (pGL4.73, 100 ng) and Firefly Luciferase (either M50 Super 8× TOPFlash or FOPFlash vector, 2 μg, kindly provided by Dr. Hanna Taipaleenmäki) using PEI (25 kD, 4 μl per well of a 1 μg/μl solution). Afterward, the cells were incubated at 37°C for 24 hrs before luciferase activity was measured using the Dual-Luciferase^®^ Reporter Assay (Promega) in a GloMax 20/20 (Promega). Calculation of sample signal was performed as following:

$$\text{signal}=\frac{\left(\text{firefly TOP sample}-\text{firefly background})/(\text{renilla TOP sample}-\text{renilla background}\right)}{\left(\text{firefly FOP sample}-\text{firefly background})/(\text{renilla FOP sample}-\text{renilla background}\right)}.$$

Subsequently, samples were normalized on values of G-2scr or G-2empty respectively.

### SHP-2 inhbition with NSC-87877

Parental G-2 cells were seeded in 6-wells (1.5 × 10^5^ per well) and grown for 48 hrs in DMEM medium with 10% FCS. The culture medium was then replaced by DMEM/10% FCS supplemented with 100 μM NSC-87877 (Merck/Millipore, #565851, stock solution: 20 mM in H_2_O) or with appropriate H_2_O-volume as mock-control. The cells were finally allowed to grow for 24 hrs, 48 hrs or 72 hrs and harvested for subsequent analyses.

### Software and statistical analyses

Numeric data were first entered in Excel (Office 2013, Microsoft) and processed with GraphPad PRISM 4.0 (GraphPad Software) for generation of the graphs as well as for statistical analyses. For the results of the qPCR, invasion- and luciferase-assays, we performed Student's *t*-tests. Fisher's exact test was employed to test the significance of the different metastasis rate in the mice. Standard error of these binominal datasets were calculated as following: SEp = sqrt [*p* (1–p) / n]. *p* values: *p ≥ 0.05; **p ≥ 0,01; ***p ≥ 0.001. Figures were generated with Inkscape (www.inkscape.org) and Photoshop (Adobe). Fluorescence and phase contrast pictures were processed with ImageJ. Confocal pictures were processed in Imaris 5.0 (Bitplane).

## SUPPLEMENTARY MATERIALS TABLES AND FIGURE


