# Supplementary material for: CEACAM1 controls the EMT switch in murine mammary carcinoma in vitro and in vivo
Source: Oncotarget. 2016 Aug 27;7(39):63730–46. doi: 10.18632/oncotarget.11650 (PMC5325399; doi:10.18632/oncotarget.11650)
Supplement: Supplementary file 1 [file oncotarget-07-63730-s001.pdf]

CEACAM1 controls the EMT switch in murine mammary carcinoma *in vitro* and *in vivo*

Supplementary Materials

Supplementary Table S1: Primers used in this study to analyse epithelial and mesenchymal gene expression signatures

Gene name	Sequences
Ceacam1	GAATCCAGTCAGCGTCAGGAG
	CCGCCAGACTTCCTGGAATAG
Cdh1	AGCCATTGCCAAGTACATCCTC
	GGCCTGTTGTCATTCTGATCTG
Epcam	GAGTCCGAAGAACCGACAAGG
	CTGATGGTCGTAGGGGCTTTC
Cdh2	AGAGCACATGCAGTGGACATC
	GGCAGTGACCGTCATCACATA
Snai1	CTGGTGAGAAGCCATTCTCCT
	CCTGGCACTGGTATCTCTTCA
Tgfb1i1	CCTCTGTGGCTCCTGCAATA
	AGCGCTCAAAGTAGCACTCG
Twist1	CGGACAAGCTGAGCAAGATTC
	TCCAGACGGAGAAGGCGTAG
Twist2	GGCCGCCAGGTACATAGAC
	GTAGCTGAGACGCTCGTGA
Vim	CGGCTGCGAGAGAAATTGC
	CCACTTTCCGTTCAAGGTCAAG
Wisp1	ACAGAGGCTGCCATCTGTGAC
	CTCGCCATTGGTGTAGCGTA
Zeb1	CACCAGAAGCCAGCAGTCAT
	CGTTCTTCTCATGGCGGTACT

Supplementary Table S2: Primary antibodies used in this study

Antibody	Spezies	Method	Dilution	Company	Cat. No.
E-Cdh (24E10)	rabbit	WB	1:2000	Cell Signaling	3195
ZO-1	rabbit	WB	1:500	Gexetex	gtx108592
Vimentin (C-20)	goat	WB	1:500	Santa Cruz	sc-7557
Ceacam1 (P2)	rabbit	WB	1:2000	A.K.H.	
Ceacam1	sheep	WB	1:2000	R&D	AF6480
β-catenin	rabbit	WB	1:2000	Sigma	C2206
p-β-catenin (S33/S37/T45)	rabbit	WB	1:1000	Cell Signaling	9561
p-β-catenin (Y86)	mouse	WB	1:500	Santa Cruz	sc-57534
Snai1	rabbit	WB	1:1000	Abcam	17732
Actin (I-19)	goat	WB	1:500	Santa Cruz	sc-1616
Tubulin (Tu20)	mouse	WB	1:500	Santa Cruz	sc-51670
SHP-2	rabbit	WB	1:1000	Cell Signaling	3397S
EpCAM (G8.8)	rat	IF	1:100	BioLegend	118201
Vimentin (C20)	goat	IF	1:400	Santa Cruz	sc-7557
Ceacam1	mouse	IF	1:100	BioLegend	134501
Ceacam1	mouse	IP	2 µg	eBioscience	13-0661
β-catenin	rabbit	IF	1:400	Sigma	C2206
Ceacam1 (PE)	mouse	FACS	1:100	BioLegend	134505
EpCAM (G8.8), APC	rat	FACS	1:100	BioLegend	118214
β-catenin	rat	IHC	1:50	R&D Systems	MAB1329
ZO-1	rabbit	IHC	1:200	Invitrogen	61-7300
SV40 large T-Ag (R15)	rabbit	IHC	1:500	W.D.	

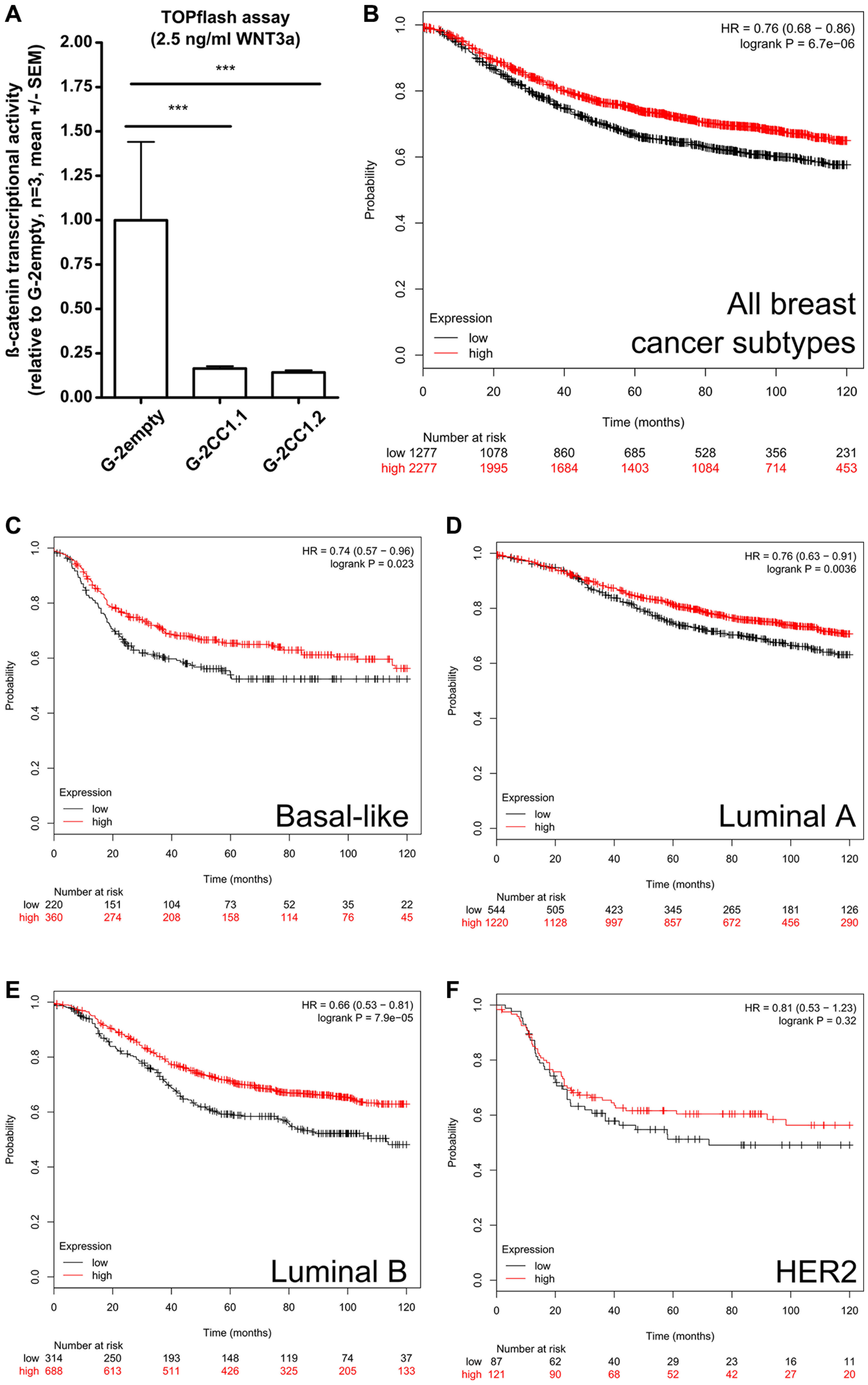

Supplementary Figure S1: Wnt-signaling activity and breast cancer patient survival are causally connected to CEACAM1-expression. (**A**) Relative transcriptional activities of β-catenin promoter assessed by TOPflash-Luciferase assays in G-2empty, G-2CC1.1 and G-2CC1.2 cells upon treatment with 10 ng/ml Wnt3a. TOPflash values were calibrated to FOPflash results and normalized to β-catenin promoter activity in G-2scr cells. Data sets are expressed as means ± SEM and were independently repeated three times in triplicate analyses. (**B**–**F**) Impact of CEACAM1 expression levels on breast cancer patient survival. This data were obtained on the public database www.kmplot.com [55, 56]. CEACAM1 expression levels inversely correlate with outcome of breast cancer independently of the tumor subtype: Kaplan-Meyer plots display survival curves (B) for all breast cancer subtypes together, (C) the basal like, (D) the luminal A, (E) the luminal B and (F) the HER2 subtypes. Parameters: Affymetrix ID: 209498_at; auto select best cutoff ON; threshold of 10 years follow up; release of the database: version 2014 (n = 4142).
